# SIK3-HDAC4 signaling pathway: the switch for transition between sleep and wakefulness

**DOI:** 10.1186/s43556-023-00128-0

**Published:** 2023-07-04

**Authors:** Yifei Tu, Fangfang Zhou, Long Zhang

**Affiliations:** 1 MOE Laboratory of Biosystems Homeostasis & Protection and Innovation Center for Cell Signaling Network, Life Sciences Institute, Zhejiang University, Hangzhou, China; 2 Institutes of Biology and Medical Science, Soochow University, Suzhou, China; 3 Cancer Center, Zhejiang University, Hangzhou, China

A recent study published in Nature by Zhou et al. [1] reported a new signaling pathway that regulates sleep in mice. This was published back-to-back with another article that found similar conclusions by identifying HDAC4 through forward genetic screening [2]. They found that the knockout of LKB1 or SIK3 proteins led to a significant decrease in the amount and delta power of non-rapid eye movement sleep (NREMS). Moreover, the phosphorylation level of HDAC4, a protein downstream of the LKB1-SIK3 axis, is also crucial for the increase in sleep. Diurnal levels and sleep can also affect the phosphorylation level of HDAC4, thus influencing the change from sleep to wakefulness [3]. These studies revealed a cascading axis of signals related to the regulation of sleep and wakefulness, referred to as the LKB1-SIK3-HDAC4 axis (Fig. 1).

**Fig. 1:**
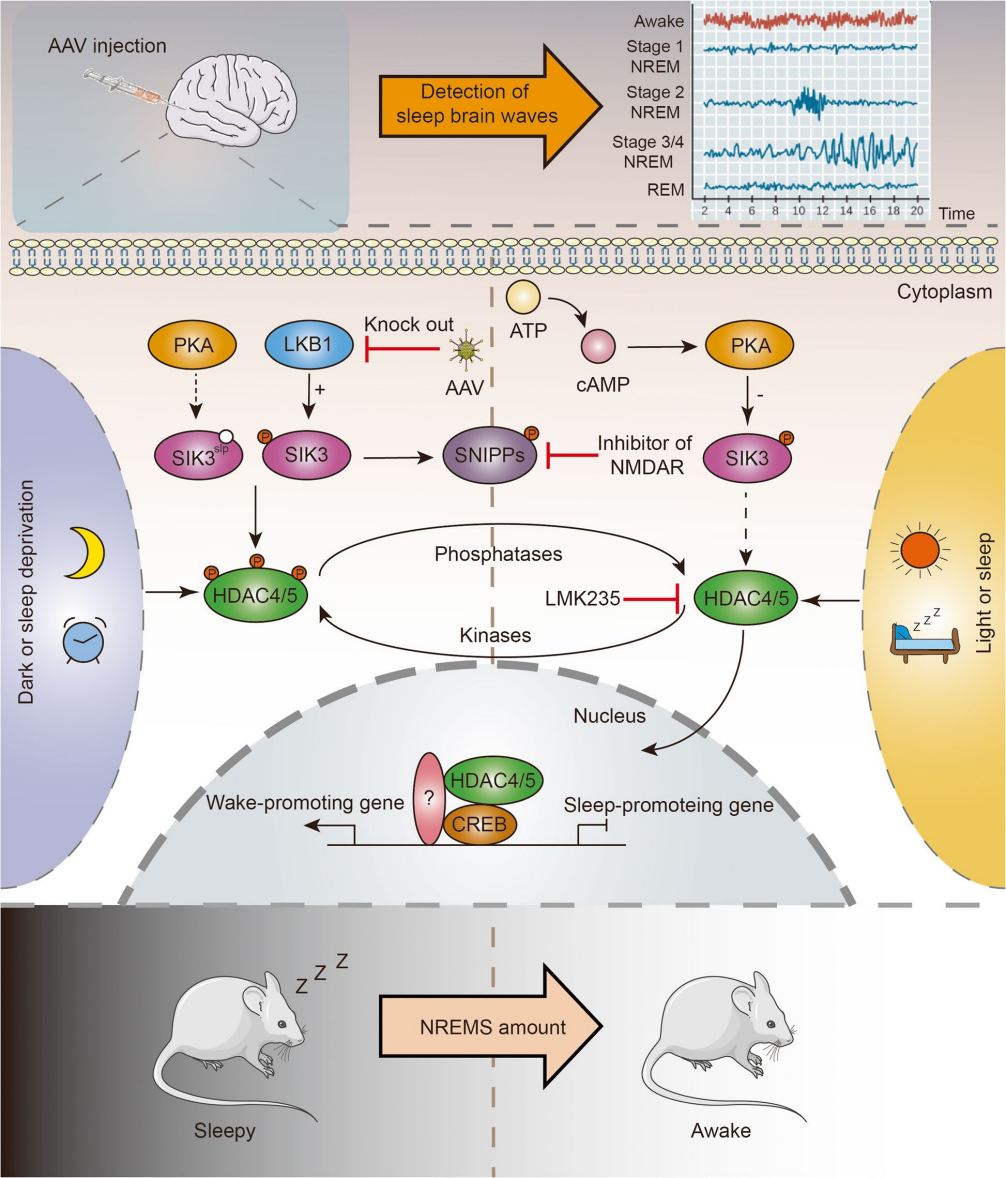
Model of LKB1-SIK3-HDAC4 signaling axis and the transcriptional regulation of non-rapid eye movement sleep in mice. Using the technique of adeno-associated virus injection and by detecting sleep brain waves, it was found that LKB1 phosphorylates SIK3, activates SIK3 activity, and phosphorylates SNIPPs and HDAC4. Furthermore, phosphorylated HDAC4 is unable to enter the nucleus and mediates the transcription of wakefulness-related genes, finally leading to wakefulness-sleep transition in mice

In mammals, sleep is a behavioral mechanism related to energy conservation; however, the specific molecular mechanisms involved in the conversion between sleep and wakefulness are unclear. Sleep need is the defintion of sleep tropism. In general, there is a positive correlation between sleep need and sleep depth and quality.

Studies have found that sleep deprivation can lead to a decline in neurological function. In addition, sleep plays a vital role in the regulation of neuroplasticity [4]. Normal physiological sleep can be divided into NREMS and rapid eye-movement sleep. The quality and depth of sleep is generally determined by monitoring the total amount of NREMS and electroencephalography during NREMS. Previous studies have reported an increased inherent sleep need in mutagenized mice through random screening of brain waves [5]. Through whole genome sequencing, it was found that drowsiness in mice was caused by the loss of part of the *SIK3* exon, resulting in an inability to be phosphorylated by protein kinase A. In contrast, phosphorylation of threonine at position 221 of SIK3 by LKB1 to activate its kinase activity can increase NREMS in mammals [2, 6]. This indicates a key role for SIK3 protein in sleep regulation. Recently, Wang et al. used proteomics and phosphorylation research methods to two different models of increased sleep need which is sleep deprivation and sleepy mice model [3]. Sleep need-related phosphorylated differential proteins downstream of SIK3 were screened and identified, such as sleep-need-index phosphoproteins (SNIPPs). The cyclic regulation of protein phosphorylation and dephosphorylation maintains a dynamic balance between sleep and wakefulness. This suggests that there are many undiscovered mechanisms in the regulation of sleep wakefulness, including the regulation of sleep need-related proteins, the specific functions and substrates of sleep demand-related proteins.

To explore this potential signaling axis, Zhou et al. [1] used adeno-associated virus (AAV)-mediated somatic genetic techniques to knock out *LKB1*, *SIK3*, *HDAC4*, and other sleep-related genes in mouse brain regions. The results showed that in *LKB1* knockout mice, the amount of NREMS and the phosphorylation level of SIK3 are significantly reduced. By constructing the SIK3 T221E mutation, which mimics SIK3 phosphorylation, NREMS levels are rectified and the NREMS delta power are rescued. This indicates that LKB1 and SIK3 are upstream and downstream of the same axis, respectively. Simultaneously, the decrease in SIK3 phosphorylation levels also leads to the enhancement of HDAC4 activity. HDAC4 expression is negatively correlated with sleep-related detection. This result was verified using wild-type and mutant HDAC4 and experimentally found that wild-type HDAC4/5 significantly reduced the delta power and the amount of NREMS, whereas when the mutated HDAC4 in the D934N model disrupted its interaction with NCoR-SMRT26 which is the transcriptional corepressor that leads HDAC4 to interact with HDAC3, it did not affect NREMS, demonstrating that HDAC4 and NCoR-SMRT26 may have a synergistic effect in regulating sleep in mice. Furthermore, in a dark environment or during periods of sleep deprivation, the phosphorylation level of HDAC4/5 is elevated, and nuclear translocation in the cerebral cortex and hypothalamus is reduced, leading to inactivation of HDAC4/5. This demonstrates that HDAC4 functions as a negative regulator of sleep-wake transition. Furthermore, through adenovirus injection, it was also found that the functional area of HDAC4 is located at the back of the hypothalamus. In addition, the LKB1-SIK3-HADC4 axis only serves to regulates the amount of sleep and has no effect on the circadian rhythm. To further investigate which transcription factors regulated by HDAC4 alter the amount of sleep, the expression of transcription factors downstream of HDAC4 were inactivated separately, which led to the identification of cAMP response element-binding protein (CREB) as being key to the alteration of sleep amount. Experimental results show that loss of CREB leads to increased sleep, and that co-expression of CREB and HDAC4 leads to a substantial reduction in daily sleep in mice and may even rescue Sleepy mice. RNA sequencing results found that HDAC4 and CREB control the regulation of the transcription of common target genes. Moreover, CHIP sequencing results revealed that HDAC4 and CREB have overlapping target genes, indicating that there is a cooperative function between CREB and HDAC4 and that they are jointly involved in gene transcription and sleep regulation.

A further paper published simultaneously performed a random mutagenesis screen and verified that mutations in *HDAC4/5* led to a reduction in NREMS [2]. In addition, the phosphorylation of HDAC4 catalyzed by SIK3 was found to promote sleep regulation by monitoring the amount of NREMS, suggesting an upstream and downstream relationship between SIK3 and HDAC4.

In conclusion, Zhou et al. [1] identified a signaling axis, the LKB1-SIK3-HDAC4 signaling axis, which regulates the sleep-wake transition. LKB1 activates SIK3 by phosphorylating the threonine site at position 221 of SIK3, which in turn activates SIK3 and phosphorylates HDAC4, inhibiting its activity and leading to an increase in the amount and delta power of NERMS. HDAC4 can translocate into the nucleus in a non-phosphorylated state and bind to transcription factors, such as CREB, to repress the transcription of sleep-promoting genes and activate wake-promoting genes. HDAC4 phosphorylation levels are also environment-dependent, with darkness and sleep deprivation both leading to increased phosphorylation levels. This paper mainly discusses the function of HDAC4 in sleep regulation and further improves the signaling axis, but some problems remain unsolved. For example, how environmental factors affect the phosphorylation level of HDAC4, the effect of circadian rhythms on this signaling pathway, and the functions of SNIPPs in this axis. Further investigation of these issues may provide further insight into the mechanics of sleep quality and may provide a new perspective on the treatment of some sleep-related disorders.

## Data Availability

Not applicable.
